# Digital Media Recruitment for Fall Prevention Among Older Chinese-American Individuals: Observational, Cross-Sectional Study

**DOI:** 10.2196/11772

**Published:** 2018-11-01

**Authors:** Nikki HT Lam, Benjamin KP Woo

**Affiliations:** 1 Northeast Ohio Medical University Rootstown, OH United States; 2 UCLA Medical Center Olive View Los Angeles, CA United States

**Keywords:** ethnic groups, falls, geriatrics, health education, social media, mobile phone

## Abstract

**Background:**

Research in fall prevention programs has increased in recent years in response to the aging demographics of the United States. To date, limited research and outreach programs have focused on ethnic minorities due to increased cost, language barriers, and cultural differences. Digital media platforms may be a cost-effective avenue to initiate fall prevention programs for minority populations.

**Objective:**

The objective of this study was to determine whether Facebook advertisements are a practical recruitment method for health education to the Chinese-speaking population.

**Methods:**

This was an observational, cross-sectional study. We uploaded a video on fall education on YouTube and initiated an advertisement campaign on Facebook that was linked to the video. The target population was older adults aged >45 years who used Facebook and were presented with the advertisement (N=1039). We recorded metrics such as the number of unique individuals reached, the number of views of the advertisement, the number of clicks, user gender and age, and traffic sources to the advertisement. Data were analyzed with descriptive statistics.

**Results:**

Our Facebook advertisement had 1087 views (1039 unique viewers). There were 121 link clicks with a click-through rate of 11.13% (121/1087). The cost per link click was approximately US $0.06. Among the viewers, 91.41% (936/1024) were females and 8.59% (88/1024) were males. In the 45-54 age group, the ad reached 50 people, with 1 link click (2.00%). In the 55-64 age group, the ad reached 572 people, with 57 link clicks (9.97%). In the ≥65 age group, the ad reached 417 people, with 63 link clicks (15.11%).

**Conclusions:**

Facebook was able to directly target the advertisement to the desired older ethnic population at a low cost. Engagement was highest among females and among those aged ≥65 years. Hence, our results suggest that Facebook can serve as an alternative platform for dissemination of health information to geriatric patients in addition to print-based and face-to-face communication.

## Introduction

Falls are highly prevalent among older adults in the United States. Every year, 2.8 million older adults are admitted to emergency departments for fall injuries, and >800,000 patients are hospitalized from fall-related injuries such as hip fracture or head injuries [1]. Falls can lead to devastating mental and physical consequences including fear for falling, low confidence, loss of independence, and premature death [2]. Furthermore, fall injuries place a tremendous economic burden on both patients and health care system [3]. As the percentage of older adults in the United States continues to increase, it is estimated that the number of fatal falls among older adults is projected to reach 100,000 per year by 2030, with an associated cost of US $100 billion [4].

In response to the aging demographics in the United States, new guidelines and outreach programs are being implemented and reformed. For example, the Centers for Disease Control and Prevention created the Stopping Elderly Accidents, Deaths, and Injuries initiative, a multiapproach program that aims to reduce fall injuries by improving screening and intervention rates at clinical settings. However, few programs were able to accommodate ethnic minorities with different culture and language backgrounds.

Minorities constitute the fastest growing segment in the rapid rise of older adults in the United States. Between 2001 and 2010, the older Asian American population increased by 145% [5]. Despite the rapidly changing demographics, research and medical services are lagging in identifying culturally influenced risk factors and in creating culturally sensitive resources [6]. Previous studies have shown that Chinese people commonly perceive falls as fatal, with low level of knowledge about fall prevention interventions, and often hide fall incidents from families and doctors [7,8]. Furthermore, Chinese-American individuals are more likely to underutilize health resources compared with Caucasians [9]. Hence, research and development of effective outreach methods is crucial in lessening the cultural and health knowledge gap among Chinese-American individuals.

Electronic health and Web-based outreach programs have shown substantial success in promoting awareness [10-14]. Popular digital marketing platforms such as Google, YouTube, and Facebook are increasingly being utilized to promote preventive medicine. A recent study showed that Facebook advertising was successful in improving the recruitment rate of older adults into a blood pressure clinical trial [10]. Another study showed that YouTube was effective in delivering dementia knowledge to older Chinese-American individuals [13]. However, few studies have investigated the role of Facebook in ethnic health outreach. Hence, we aimed to examine (1) whether Facebook advertising strategies are cost effective; (2) the ability of Facebook in targeting fall prevention and education to the Chinese geriatric populations; and (3) engagement among the targeted geriatric populations on Facebook compared with other social media platforms such as YouTube. We hope that this study can provide insights into the use of social media marketing in public health outreach for the geriatric populations.

## Methods

### Facebook Advertisement

We uploaded a recording of a 37-minute-long video of a medical education talk show at the radio station KMRB AM1430 in Los Angeles on YouTube in May 2016 [15]. The show was conducted entirely in Cantonese; contents included prevalence, risk factors, prevention methods, and cultural factors and misconceptions about falls. An advertisement was then initiated on Facebook for 48 hours in September 2017, linking interested individuals to the video. The advertisement included a still of the video, an 11-character title, and a 23-character text body in traditional Chinese. The title can be translated into English as “What are the factors for loss of balance and falls among older adults?”

### Participants

The population sampled in this study included all viewers who saw the Facebook advertisement on Web during the 48-hour advertising campaign. According to the current literature, falls and related injuries are most prevalent in adults aged >65 years [1]. Therefore, we set the age of our target audience to be adults aged ≥45 years to include individuals who were beginning to enter the high-risk ages. These details were included as part of the initial Facebook advertisement registration process.

### Statistical Analysis

Data on the advertisement campaign were obtained via Facebook analytics. Parameters included the number of individuals reached (defined as unique advertisement viewers), the number of impressions (defined as the total number of views of the advertisement), the number of engagements (defined as the number of likes, clicks, or shares), the click-through rate (clicks/impressions), and the cost per click. In addition, demographic information including gender, age, and traffic sources was recorded. Data obtained via Facebook analytics were further confirmed using data directly obtained from the video link via YouTube analytics during the 48-hour campaign. Data were analyzed using descriptive statistics.

## Results

### Overall Ad Performance

Overall, the Facebook advertisement had 1087 impressions, of which 95.58% (1039/1087) views were by unique individuals (reach). Of 125 engagements recorded by Facebook analytics, 121 were link clicks, 3 were post reactions, and 1 was a post share. The click-through rate (clicks/impressions) was 11.13% (121/1087). The total cost of the advertisement for 48 hours was US $6.82. The cost per 1000 impressions was US $6.27, and the cost of the ad per link click was US $0.06.

### Ad Performance by Gender, Age, and Traffic Sources

Table 1 presents the performance of the Facebook advertisement by different ages and gender. The advertisement reached 91.41% (936/1024) female viewers, with 112 link clicks (click-through rate, 11.97%). Conversely, the advertisement reached 8.59% (88/1024) male viewers, with 9 link clicks (click-through rate, 10.23%). Hence, the cost per link click among female viewers was US $0.05, while the cost per link click among male viewers was US $0.07.

**Table 1:** Descriptive summary of Facebook advertisement performance by gender and ages.

Variable		Reach, n (%)	Link clicks, n (%)	Cost per link click
**Gender**				
	Female	936 (91.41)	112 (11.97)	US $0.05
	Male	88 (8.59)	9 (10.23)	US $0.07
**Age (years)**				
	45-54	50 (4.81)	1 (2.00)	US $0.23
	55-64	572 (55.05)	57 (9.97)	US $0.07
	≥65	417 (40.13)	63 (15.11)	US $0.04

In the 45-54 years age group, the ad reached 4.81% (50/1039) viewers, with 1 (2.00%) link click. In the 55-64 years age group, the ad reached 55.05% (572/1039) people, with 57 (9.96%) link clicks. In the ≥65 years age group, the ad reached 40.13% (417/1039) people, with 63 (15.11%) link clicks. The costs per link click were US $0.23, US $0.07, and US $0.04 for each group, respectively.

In the 45-54 years age group, 38 females and 12 males were reached; in the 55-64 years age group, 523 females and 43 males were reached; and in the ≥65 years age group, 375 females and 33 males were reached. There were 15 impressions with unknown gender. Only 1 female and no males clicked the advertisement in the 45-54 years age group; 52 females and 5 males clicked the advertisement in the 55-64 years age group; and 59 females and 3 males clicked in the ≥65 years age group.

With regards to device usage, the advertisement reached 99.62% (1035/1039) mobile device users, resulting in all 121 of the link clicks recorded. Only 4 users were reached through desktop devices, and there were no link clicks via desktop devices.

### Comparison With YouTube Data

During the first year when the same educational video was uploaded on YouTube, YouTube data showed 588 views with an average view duration of 27% (9.82/37.1 minutes) of the total video length. Mobile phones accounted for 52.2% (307/588) of the views.

In comparison, during the 48-hour advertisement run, the video had 75 views on YouTube, of which 66 viewers arrived from external websites. The total watch time was 440 minutes, and the average view duration was 16% (5.87/37.1 minutes) of the total video length. Data on audience retention revealed that 13% viewers (10/75 of viewers during this time period) finished all 37.1 minutes of the video. Mobile phones accounted for 85% (64/75) views.

## Discussion

### Principal Findings

This study demonstrates that Facebook was able to attract viewers to Web-based health education resources at low cost, as well as correctly target the advertisement at the desired older ethnic population. Engagement and cost-effectiveness were highest among those aged ≥55 years. This ultimately suggests that older Asian individuals may benefit from targeted preventive health and educational interventions delivered through Facebook.

One advantage of using Facebook is its ability to directly target a certain demographic group; in our study, we were able to target our advertisement on fall education toward Chinese-American older adults with an above-average click-through rate (11.13% vs 0.90%) and a lower cost per click (US $0.06 vs US $1.72) compared with the Health and Medical Industry average [16]. On comparing with similar studies that were instead targeted toward younger adults, click-through rate of 11.13% (121/1087) of the older adults was almost 4 times as high as that of younger adults engaged with digital media advertising in other health outreach studies [17]; this suggests that older Chinese-American individuals have a relatively high interest in falls-related education, and they may be more receptive to remote health education compared with younger adults. In addition, this method of outreach is highly cost effective. Hence, our results suggest that Facebook could be an alternative way to reach this population despite the common belief that the geriatric population does not use social media.

A few other noteworthy patterns were also observed in this study. First, the advertisement disproportionately reached a very large number of female viewers compared with male viewers. Similar results have been noted in several previous studies on Facebook advertisements, which could be attributed to higher rates of Facebook usage by females across all ages [18-20]. In addition, the click-through rate for female viewers was slightly higher than that for male viewers. Moreover, click-through rates improved with the increasing age, causing cost efficiency to increase (as cost-per-click decreases) with the increasing age. The higher click-through rate among females and among older adults aged ≥65 years also suggest that these groups are more interested in obtaining health education through social media compared with males and other age groups. Furthermore, mobile devices accounted for a large amount of traffic through the linked advertisement.

Comparison of the data received from Facebook and the data gleaned from YouTube analytics during the 48-hour run of the advertisement revealed that the Facebook advertisement was able to increase traffic to the YouTube video. During the first year after uploading the video on YouTube, there were 588 views. During the 48 hours analyzed, there were 75 new views of the video, indicating that the advertisement was successful in directing traffic. Interestingly, the average view duration dropped from 9.82 minutes during the first year to 5.87 minutes during the 48 hours. We speculate that this may be due to the fact that many Facebook users were not actively seeking out information on fall prevention when they came across the advertisement and, thus, may have a shorter engagement time than those who were actively searching for such information. However, the average view duration recorded from this advertisement coincides with the reported median engagement time of 6 minutes for Web-based educational videos, suggesting that the video was able to capture viewers’ attention [21].

### Limitations

Limitations of this study include the inability of researchers to assess knowledge before and after viewing of the video. As such, the efficacy of the video in providing information on falls remains unknown. Additionally, because of the anonymity of internet users, as stated previously, there is no way of knowing the motivation of viewers for watching the video or their actual socioeconomic status. Furthermore, the study video was conducted in Cantonese; therefore, the audience reached was likely a selected group of Chinese-American individuals who are fluent in that language. Moreover, the advertisement only ran for 2 days, which qualifies as a short campaign. It remains unknown whether a longer campaign would have better or worse impact on the overall views of the video. Finally, the engagement time for the video was short. In the future, the video should be shortened with more visual aids and focused content to improve viewers’ engagement.

### Conclusions and Future Direction

Future studies should explore how to optimize advertisement descriptions to maximize the click-through rate. Owing to our result showing the high prevalence of female users in the older ethnic Facebook user population, we suggest that Facebook may provide an attractive platform for future distribution of information regarding women’s health. In addition, future studies will likely have to address the increasing prevalence of mobile devices in exploring Web-based health care resources, as demonstrated by this study.

In this study, we were successful in engaging users; however, more research is needed in determining how to receive feedback regarding what this population may need in terms of health education information. Most viewers were older individuals belonging to an ethnic population; however, the very anonymity that attracts internet and social media users also makes it difficult for researchers to determine why this patient population is watching health education videos and to figure out the type of help they need. The question of how to bridge this gap between remote-based tools, internet anonymity, and clinical care is the next problem to solve in the internet and social media health information delivery research.
